# Recurrence after piecemeal hot-snare endoscopic mucosal resection of 10–20-mm nonpedunculated colorectal polyps: a multicenter cohort study

**DOI:** 10.1055/a-2563-1606

**Published:** 2025-05-19

**Authors:** Michiel H. J. Maas, Yark Hazewinkel, Jochim S. Terhaar Sive Droste, Ruud W. M. Schrauwen, Adriaan C. Tan, Parweez Koehestanie, Mariëtte C. A. van Kouwen, Peter D. Siersema

**Affiliations:** 1 Department of Gastroenterology and Hepatology, Radboud University Medical Center, Nijmegen, Netherlands; 2 Department of Gastroenterology and Hepatology, Ter Gooi Hospital, Hilversum, Netherlands; 3 Department of Gastroenterology and Hepatology, Jeroen Bosch Hospital, 's-Hertogenbosch, Netherlands; 4 Department of Gastroenterology and Hepatology, Hospital Bernhoven, Uden, Netherlands; 5 Department of Gastroenterology and Hepatology, Canisius Wilhelmina Hospital, Nijmegen, Netherlands; 6 37226 Department of Gastroenterology and Hepatology, Bravis Hospital, Roosendaal, Netherlands; 7 Department of Gastroenterology and Hepatology, Erasmus MC University Medical Center, Rotterdam, Netherlands

## Abstract

**Background:**

Guidelines are equivocal on the need for early surveillance colonoscopy (ESC) after piecemeal endoscopic mucosal resection (pEMR) of 10–20-mm nonpedunculated colorectal polyps (NPCPs). This study assessed recurrence rates and associated factors at ESC following hot-snare pEMR of 10–20-mm NPCPs.

**Methods:**

A retrospective, multicenter cohort study was performed at five hospitals in the Netherlands. Patients undergoing pEMR of 10–20-mm NPCPs (2014–2021) and referred for ESC (range 3–9 months) were included. The primary outcome was recurrence rate at ESC. Secondary outcomes included scar identification rates, both overall and at tattooed sites. A mixed-effects model was used to identify factors associated with recurrence.

**Results:**

389 patients undergoing pEMR of 426 NPCPs 10–20 mm (median 15 mm, interquartile range 12.8–20.0 mm) were included. Overall, 262 scars (61.5%; 95%CI 56.8–66.0) and 81.6% of tattooed sites were identified at ESC. The recurrence rate was 35/426 (8.2%; 95%CI 6.0–11.2) overall and 35/262 (13.4%; 95%CI 9.8–18.0) when the scar was identified. Median recurrence size was 5 mm, without high grade dysplasia. No NPCP characteristics were associated with recurrence.

**Conclusions:**

This real-world study found a substantial recurrence rate after hot-snare pEMR of NPCPs sized 10–20mm at ESC. ESC scar identification was moderate but improved with tattoo placement. Although early surveillance could be considered to avoid missing recurrence, the small recurrence size and absence of high grade dysplasia suggest that modestly extending the interval beyond that of our study may still allow timely detection of recurrences and metachronous lesions.

## Abstract (de)

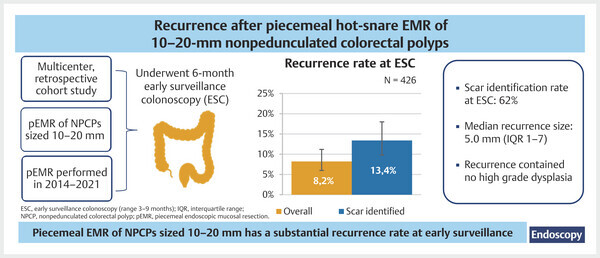

## Introduction

Colorectal polyps are potential precursor lesions of colorectal cancer (CRC), and endoscopic resection reduces CRC-related mortality 1 . The recommended method of endoscopic removal depends, among others, on polyp morphology and size. According to guidelines of the European Society of Gastrointestinal Endoscopy (ESGE) and the US Multi-Society Task Force on Colorectal Cancer (USMSTF), endoscopic mucosal resection (EMR) or endoscopic submucosal dissection (ESD) are advised for the removal of nonpedunculated colorectal polyps (NPCPs) sized ≥20 mm 2 3 . However, recommendations for polyps sized 10–20 mm are less clear 4 5 . Nevertheless, a recent meta-analysis found that conventional hot-snare EMR with submucosal injection is the most commonly performed resection technique for lesions sized 10–20 mm 6 .

Despite the effectiveness of EMR, there remains a risk of recurrent or residual lesions, particularly with piecemeal EMR (pEMR), which has an increased risk of recurrence compared with en bloc resections 2 7 8 . While high-quality studies investigating the incidence, predictors, and preventative techniques for NPCPs sized ≥20 mm are increasingly being performed, studies specifically addressing recurrence rates and potential predictors for resecting NPCPs sized 10–20 mm remain scarce 8 9 10 11 . Previous studies reported recurrence or incomplete resection rates of up to 20.4% for EMRs of NPCPs sized 10–20 mm 4 6 7 . However, these studies frequently pooled data of various polyp sizes and resection techniques, obscuring a clear understanding of the risk of recurrence after pEMR in this specific size category.

Therefore, the aim of this retrospective, multicenter cohort study was to evaluate the recurrence rate, associated factors, and post-EMR scar identification rate in patients undergoing early surveillance colonoscopy (ESC) after pEMR of NPCPs sized 10–20 mm.

## Methods

### Study design

This retrospective, multicenter cohort study was conducted at five hospitals in the Netherlands: two teaching hospitals and three non-teaching hospitals. The Medical Ethical Committee Oost-Nederland waived the requirement for ethical approval according to Dutch law (Dutch Medical Research Involving Human Subjects Act), reference 2021–13058, issued on July 15, 2021, due to the retrospective design of the study and negligible risk for patients. Consequently, the need for informed consent procedures was waived. Approval from each participating study site was obtained, ensuring adherence to local standards and protocols. The study was reported according to the Strengthening the Reporting of Observational Studies in Epidemiology (STROBE) statement.

### Patients

Historic electronic medical record data from patients at each study site underwent review by a study team member following the application of specific search terms, including “piecemeal,” “endoscopic mucosal resection,” “EMR,” or “pEMR,” to identify cases from the endoscopy patient cohorts. Adult patients were included in the cohort if they underwent hot-snare EMR with submucosal injection in a piecemeal fashion (minimum of two pieces per resection), of an NPCP sized 10–20 mm, and were reported as complete resections, between January 1, 2014, and December 31, 2021, and subsequently underwent surveillance colonoscopy within 3–9 months after primary resection. This timeframe aligns with the national 2013 Dutch post-polypectomy guidelines and 2013 ESGE guidelines, which recommended early repeat colonoscopy 6 months after pEMR 12 13 . Although NPCPs sized 20 mm are generally considered large NPCPs in polypectomy guidelines 2 3 , NPCPs reported as 20 mm were included in the study cohort to address potential terminal digit bias, which frequently leads to an overrepresentation of reported lesion sizes ending in 0 or 5 14 . In addition, patient records were re-reviewed through August 31, 2024, to collect additional information from surveillance colonoscopies conducted after ESC (SC2). Patients with polyposis syndrome or known/suspected inflammatory bowel disease were excluded.

### Study variables collected

Data collection was performed at each study site using a standardized electronic data collection form in CastorEDC (Ciwit B.V., Amsterdam, The Netherlands) with prespecified criteria for coding. Study variables included patient demographics, study site, primary pEMR characteristics (including year of resection, level of training [nurse endoscopist, junior or senior endoscopist]), and whether the endoscopist was considered an EMR expert, defined as performing an average of ≥30 EMRs or ESDs per year based on historical data. Lesion location was categorized per colonic segment and dichotomized to proximal colon (defined as proximal to the splenic flexure) or distal colon (defined as distal to the splenic flexure). Additional variables included lesion size, Boston Bowel Preparation Scale (BBPS) score, use of adjuvant modalities, presence of intraprocedural bleeding, placement of a tattoo, and histopathological diagnosis based on the Vienna criteria 15 .

Surveillance colonoscopy characteristics included interval to surveillance colonoscopy, level of training, identification of the post-EMR scar, identification of placed tattoos, BBPS score, and histopathological diagnosis of recurrence or biopsies from the post-EMR site. Data were collected on the presence of recurrence at the identified scar, or advanced neoplasia, defined as lesions ≥10 mm, or those containing high grade dysplasia (HGD) at SC2. The end of follow-up was defined as the detection of recurrence at ESC or completion of SC2.

### Primary and secondary outcomes

**Fig. 1:**
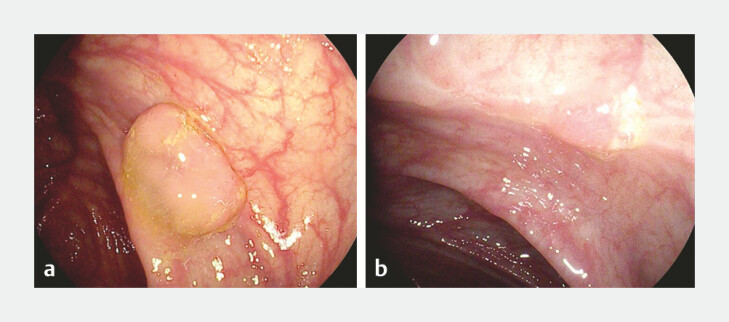
Local recurrence after piecemeal endoscopic mucosal resection (pEMR) of nonpedunculated colorectal polyps (NPCP). **a** A 15-mm NPCP located in the cecum, which was resected by pEMR. **b** Scar identified with recurrence at early surveillance colonoscopy.

The primary outcome was the recurrence rate at ESC after pEMR of 10–20-mm NPCPs. Recurrence rates were reported both for all colonoscopies and for cases where the scar was identified. Recurrence was defined as the presence of macroscopic or microscopic tissue at the post-EMR site, confirmed by histopathological diagnosis ( Fig. 1 ). Secondary outcomes included the rate of identified post-EMR scars during ESC, variables potentially associated with recurrence and scar identification, and the presence of recurrence at the identified scar or advanced neoplasia within the same colonic segment as the primary resection (cecum, ascending colon, transverse colon, descending colon, sigmoid colon, or rectum) during SC2.

### Statistical analysis

Patient and procedural characteristics were summarized using descriptive statistics. Categorical variables were presented as absolute frequencies and percentages, while continuous variables were reported as means with SD or medians with interquartile ranges (IQRs), as appropriate. Inferential comparisons were conducted using independent *t* tests, Mann–Whitney *U* tests, or chi-squared tests, as appropriate. Wilson Score Interval was used to calculate 95%CIs where applicable.

Potential associations with recurrence and scar identification were assessed using mixed-effects binary logistic regression models to account for clustering. Study site was included as a random effect. Variables with a *P* value ≤0.20 in the univariable analysis for the outcome of interest were included in the multivariable model. Backward elimination was used, sequentially removing the variable with the highest *P* value until only variables with *P* ≤ 0.05 remained, while maintaining a maximum of one covariate per 10 events of the outcome of interest.

Cumulative incidence functions were used to calculate the incidence of recurrence or advanced neoplasia at SC2 for cases with and without scar identification at ESC. To minimize potential bias related to the surveillance interval, cases with synchronous lesions sized ≥20 mm at ESC were excluded. Competing risks analysis was performed using Gray’s test to compare the incidence of recurrence or advanced neoplasia between cases with and without scar identification at ESC. Competing events included death, canceled SC2 due to comorbidities or advanced age, and transfer of care to another hospital.

The level of statistical significance was set at *P* < 0.05, unless otherwise specified. No imputation of missing data was performed. Statistical analysis was performed using Statistical Package for Social Sciences program, version 29 (IBM Corp., Armonk, New York, USA) and R Statistical Software (v.4.1.3; packages *“cmprsk”* and *“binom”* ; R Foundation for Statistical Computing, Vienna, Austria).

## Results

### Patient and procedural characteristics

A total of 389 patients with 426 NPCPs sized 10–20 mm who underwent pEMR and ESC were included in the cohort. The median age was 68.0 years (IQR 63.0–72.0), and 219 patients (56.3%) were male. NPCPs were resected by 103 endoscopists, with a median of 3 (IQR 1–6) pEMRs procedures per endoscopist. The median NPCP size was 15.0 mm (IQR 12.8–20.0 mm). Most NPCPs (80.3%; 342/426) were located in the proximal colon, and were resected by senior endoscopists in 367 cases (86.2%) and by EMR experts in 98 cases (23%). Adjuvant margin thermal ablation (MTA) with snare-tip soft coagulation was performed for 67 NPCPs (15.7%) and argon plasma coagulation was used for 25 (5.9%). A tattoo was placed at the primary pEMR site of 76 NPCPs (17.8%). Additional characteristics are presented in Table 1 (see also **Table 1s–3s** , and **Fig. 1s** in the online-only Supplementary material).

**Table:** **Table 1** Patient, polyp, and primary and early surveillance colonoscopy characteristics.

	Overall	No recurrence at ESC	Recurrence at ESC
Patient information	**n = 389**	**n = 355**	**n = 34**
Age, median (IQR), years	68.0 (63.0–72.0)	68.0 (63.0–72.0)	68.0 (63.0–73.3)
Sex			
Male	219 (56.3)	199 (56.1)	20 (58.8)
Female	170 (43.7)	156 (43.9)	14 (41.2)
Family history ^1^			
Positive family history for CRC ^2^	30 (8.9)	28 (9.2)	2 (6.1)
Lynch syndrome	8 (2.4)	8 (2.6)	0 (0)
Reason for colonoscopy			
Non-FIT screening	7 (1.8)	7 (2.0)	0 (0)
FIT+ screening	159 (40.9)	143 (40.3)	16 (47.1)
Surveillance	120 (30.8)	109 (30.7)	11 (32.4)
Symptomatic	49 (12.6)	47 (13.2)	2 (5.9)
Scheduled EMR procedure	49 (12.6)	44 (12.4)	5 (14.7)
Other	5 (1.3)	5 (1.4)	0 (0)
NPCP and procedural information	**n = 426**	**n = 391**	**n = 35**
Lesion size, median (IQR), mm	15.0 (12.8–20.0)	15.0 (12.0–20.0)	15.0 (15.0–18.0)
Location of primary lesion			
Proximal colon ^3^	342 (80.3)	318 (81.3)	24 (68.6)
Distal colon	84 (19.7)	73 (18.7)	11 (31.4)
pEMR performed by			
Senior endoscopist	367 (86.2)	338 (86.4)	29 (82.9)
Junior endoscopist	49 (11.5)	43 (11.0)	6 (17.1)
Nurse endoscopist	10 (2.3)	10 (2.6)	0 (0.0)
EMR expert ^4^	98 (23.0)	93 (23.8)	5 (14.3)
No. of pieces per pEMR, median (IQR) ^5^	2.0 (2.0–3.0)	2.0 (2.0–3.0)	3.0 (2.0–3.3)
Intraprocedural bleeding, yes	55 (12.9)	54 (13.8)	1 (2.9)
Post-EMR clip placement, yes	116 (27.2)	108 (27.6)	8 (22.9)
Use of MTA			
STSC	67 (15.7)	64 (16.4)	3 (8.6)
APC	25 (5.9)	24 (6.1)	1 (2.9)
Tattoo placed, yes	76 (17.8)	67 (17.1)	9 (25.7)
ESC information	**n = 426**	**n = 391**	**n = 35**
Time to ESC, median (IQR), weeks	27.0 (23.8–31.0)	27.0 (24.0–31.0)	27.0 (20.0–34.0)
ESC performed by			
Senior endoscopist	314 (73.7)	285 (72.9)	29 (82.9)
Junior endoscopist	76 (17.8)	70 (17.9)	6 (17.1)
Nurse endoscopist	36 (8.5)	36 (9.2)	0 (0)
EMR expert ^4^	97 (22.8)	88 (22.5)	9 (25.7)
BBPS of segment of post-pEMR site ^6^			
3	248 (70.3)	226 (68.9)	22 (88.0)
2	98 (27.8)	95 (29.0)	3 (12.0)
1	6 (1.7)	6 (1.8)	0 (0)
0	1 (0.3)	1 (0.3)	0 (0)
Biopsy of post-pEMR scar	23 (8.8)	18 (7.9)	5 (14.3)
Data are presented as n (%) unless otherwise stated. APC, argon plasma coagulation; BBPS, Boston Bowel Preparation Score; CRC, colorectal cancer; ESC, early surveillance colonoscopy; ESD, endoscopic submucosal dissection; FIT, fecal immunochemical test; IQR, interquartile range; MTA, margin thermal ablation; NPCP, nonpedunculated colorectal polyp; (p)EMR, (piecemeal) endoscopic mucosal resection; STSC, snare-tip soft coagulation. ¹Family history was not reported in 53 patients (no recurrence group n = 52; recurrence group n = 1). ^2^ Defined as one first-degree relative diagnosed before the age of 50 years, or one first-degree relative diagnosed aged 50–70 years and one second-degree relative diagnosed before the age of 70 years, or two or more first-degree relatives diagnosed aged 50–70 years. ^3^ Proximal colon was defined as all segments proximal to the splenic flexure. ^4^ EMR expert was defined as an endoscopist with a minimum of 30 performed EMRs or ESDs per year on average, based on historic data. ^5^ Median calculated on the total number of cases (n = 93) in which number of pieces per resection was reported. ^6^ BBPS score was not reported for 73 patients (no recurrence group n = 63; recurrence group n = 10).			

### Scar identification and recurrence rates at ESC

ESC was performed after a median interval of 27.0 weeks (IQR 23.8–31.0) following the primary pEMR ( Table 1 ). The post-pEMR scar was identified in 262 of 426 colonoscopies performed at ESC (61.5%; 95%CI 56.8–66.0) ( Table 2 ). Scar identification rate was 81.6% (62/769; 95%CI 71.4–88.7) for tattooed resection sites compared with 57.1% (200/350; 95%CI 51.9–62.2) for non-tattooed resection sites ( *P* <0.001). Overall, recurrence at the post-pEMR site was observed in 35 of 426 NPCPs (8.2%; 95%CI 6.0–11.2), with a median recurrence size of 5 mm (IQR 1–7). Recurrence rates were 9.0% (26/289; 95%CI 6.2–12.9) for adenomas and 7.0% (9/128; 95%CI 3.7–12.8) for serrated lesions. Recurrence was observed in 4 of 92 lesions treated with MTA (4.3%; 95%CI 1.7–10.7), and in 31 of 334 lesions not treated with MTA (9.3%; 95%CI 6.6–12.9). Recurrent lesions contained no HGD and had no HGD in their primary resection. In colonoscopies with a post-pEMR scar identified (n = 262), recurrences were observed in 35 (13.4%; 95%CI 9.8–18.0). Additional results are reported in Table 2 , and **Tables 4s–8s** .

**Table:** **Table 2** Overall recurrence rates, recurrence rates per adenomatous or serrated lesions, and scar identification rates at early surveillance colonoscopy.

	Early surveillance colonoscopy
Overall recurrence	35/426 (8.2) [6.0–11.2]
Recurrence of adenomas	26/289 (9.0) [6.2–12.9]
Recurrence of serrated lesions ^1^	9/128 (7.0) [3.7–12.8]
Recurrence of NPCPS with HGD	0/35 (0) [0.0–9.9]
Recurrence of other NPCPs ^2^	0/9 (0) [0.0–29.9]
Recurrence of NPCPs 16–20 mm	10/171 (5.8) [3.2–10.4]
Recurrence of NPCPs 10–15 mm	25/255 (9.8) [6.7–14.1]
Recurrence of NPCPs treated with MTA	4/92 (4.3) [1.7–10.7]
Recurrence of NPCPs treated without MTA	31/334 (9.3) [6.6–12.9]
Recurrence size, ^3^ median (IQR), mm	5.0 (1.0–7.0)
Identification of post-pEMR scar	262/426 (61.5) [56.8–66.0]
Identification of tattooed resection sites	62/76 (81.6) [71.4–88.7]
Identification of non-tattooed resection sites	200/350 (57.1) [51.9–62.2]
Overall recurrence of cases with scar identification	35/262 (13.4) [9.8–18.0]
Data are presented as n/N (%) [95%CI] unless otherwise stated. 95%CI was calculated with Wilson Score Interval. HGD, high grade dysplasia; IQR, interquartile range; NPCP, nonpedunculated colorectal polyp; MTA, margin thermal ablation. ^1^ Serrated lesions included sessile serrated lesions (n = 105), hyperplastic polyps (n = 17), and traditional serrated adenomas (n = 6). ^2^ Other lesions (n = 9) included NPCPs with indefinite or missing histopathologic diagnosis. ^3^ Size of recurrence was reported in 19 of 30 cases with macroscopic recurrence.	

### Associations with recurrence and scar identification at ESC

Our mixed-effects binary regression model analysis found no variables significantly associated with recurrence at ESC in either univariable or multivariable analysis ( Table 3 ). The mixed-effects model found that tattoo placement (odds ratio [OR] 3.50; 95%CI 1.81–6.77), a segmental BBPS score of 3 vs. ≤2 at ESC in the same segment as the primary resection (OR 2.07; 95%CI 1.28–3.37), and post-EMR clip placement (OR 2.13; 95%CI 1.28–3.56) were independently and statistically significantly associated with scar identification at ESC ( Table 4 ).

**Table:** **Table 3** Binary logistic mixed-effects model regression results for variables associated with local recurrence at early surveillance colonoscopy.

	Univariable analysis		Multivariable analysis ^1^	
	OR (95%CI)	P	OR (95%CI)	P
Age at primary resection, years	1.02 (0.98–1.08)	0.35		
Interval to ESC	1.00 (0.95–1.06)	0.89		
Female sex	0.81 (0.40–1.64)	0.55		
Reason for colonoscopy (ref: FIT+ screening)				
Surveillance	0.87 (0.39–1.91)	0.72		
Symptomatic	0.33 (0.08–2.90)	0.20		
Scheduled EMR procedure	0.97 (0.33–2.90)	0.96		
Positive family history for CRC ^2^ (ref: no family history)	0.63 (0.14–2.80)	0.55		
Primary lesion in proximal colon (ref: distal colon) ^3^	0.54 (0.25–1.16)	0.11	0.55 (0.26–1.20)	0.14
Primary pEMR performed by junior endoscopist (ref: senior endoscopist)	1.28 (0.48–3.46)	0.62		
pEMR performed by EMR expert ^4^	0.48 (0.18–1.33)	0.16	0.50 (0.18–1.38)	0.18
No. of pEMR performed per endoscopist (ref: bottom tertile, 1–5 pEMRs) ^5^				
Mid tertile (6–8 pEMRs)	0.78 (0.32–1.89)	0.58		
Highest tertile (>8 pEMRs)	0.73 (0.32–1.68)	0.46		
Primary lesion size, mm	1.00 (0.91–1.10)	0.96		
No. of pieces per resection ^6^	1.37 (0.55–3.37)	0.49		
Intraprocedural bleeding	0.17 (0.23–1.28)	0.09		
Post-pEMR clip placement	0.75 (0.31–1.80)	0.51		
Adjuvant use of MTA	0.42 (0.14–1.24)	0.12	0.44 (0.15–1.29)	0.13
STSC	0.46 (0.13–1.57)	0.21		
APC	0.42 (0.05–3.25)	0.41		
Serrated lesion	0.81 (0.37–1.80)	0.61		
HGD	0.68 (0.09–5.43)	0.72		
APC, argon plasma coagulation; CRC, colorectal cancer; ESC, early surveillance colonoscopy; ESD, endoscopic submucosal dissection; FIT, fecal immunochemical test; HGD, high grade dysplasia; MTA, margin thermal ablation; OR, odds ratio; (p)EMR, (piecemeal) endoscopic mucosal resection; STSC, snare-tip soft coagulation. ^1^ Study site was used as a random effect in this mixed-effects model to account for site-level clustering. Variables with a *P* value ≤0.20 in the univariable analysis were included in the multivariable analysis. Backward elimination was applied, removing variables sequentially by *P* value until only those with *P* ≤ 0.05 remained. As no variables met this final inclusion criterion, the first step of the model is presented. Due to the limited number of recurrence cases, the number of variables was restricted to a maximum of three in the model. ^2^ Analysis was performed on the 370 cases with data on family history. ^3^ Proximal colon defined as all segments proximal to the splenic flexure. ^4^ EMR expert was defined as an endoscopist with a minimum of 30 performed EMRs or ESDs per year on average, based on historic data. ^5^ Tertiles of performed pEMRs per endoscopist were categorized based on the 33rd and 66th percentile. ^6^ Analysis was performed on the 93 cases with data on the number of pieces per resection.				

**Table:** **Table 4** Binary logistic mixed-effects model regression results for variables associated with scar identification at early surveillance colonoscopy.

	Univariable analysis		Multivariable analysis ^1^	
	OR (95%CI)	*P*	OR (95%CI)	*P*
Interval to ESC, weeks	1.00 (0.97–1.04)	0.89		
Primary lesion size, mm	1.09 (1.03–1.15)	0.003		
Tattoo placed, yes	3.16 (1.69–5.90)	<0.001	3.50 (1.81–6.77)	<0.001
BBPS score of 3 at ESC ^2^ (ref: BBPS of ≤2)	1.95 (1.22–3.11)	0.005	2.07 (1.28–3.37)	0.003
ESC performed by (ref: senior endoscopist)				
Junior endoscopist	0.69 (0.37–1.29)	0.25		
Nurse endoscopist	0.66 (0.32–1.38)	0.27		
ESC performed by EMR expert	1.18 (0.73–1.92)	0.51		
ESC performed by the same endoscopist as the primary pEMR	0.99 (0.61–1.61)	0.96		
Location of primary lesion (ref: rectum)				
Cecum	0.998 (0.42–2.35)	0.99		
Ascending colon	0.60 (0.26–1.37)	0.22		
Hepatic flexure	0.36 (0.12–1.06)	0.06		
Transverse colon	0.47 (0.20–1.12)	0.09		
Splenic flexure	1.21 (0.20–7.42)	0.84		
Descending colon	0.83 (0.26–2.71)	0.76		
Sigmoid	0.88 (0.28–2.75)	0.83		
Post-pEMR clip placement	1.60 (1.01–2.54)	0.047	2.13 (1.28–3.56)	0.004
Adjuvant use of MTA	1.52 (0.92–2.51)	0.10		
Serrated lesion	1.23 (0.79–1.91)	0.36		
HGD in primary lesion	0.94 (0.32–2.72)	0.90		
BBPS, Boston Bowel Preparation Scale; ESC, early surveillance colonoscopy; HGD, high grade dysplasia; MTA, margin thermal ablation; OR, odds ratio; (p)EMR, (piecemeal) endoscopic mucosal resection. ^1^ Study site was used as a random effect in this mixed-effects model to account for site-level clustering. Variables with a *P* value ≤0.20 in the univariable analysis were included in the multivariable analysis. Backward elimination was applied, removing variables sequentially by *P* value until only those with *P* ≤ 0.05 remained. ^2^ 73 cases with missing values were not included in the analysis.				

### Recurrence or advanced neoplasia at surveillance colonoscopy after ESC

**Fig. 2:**
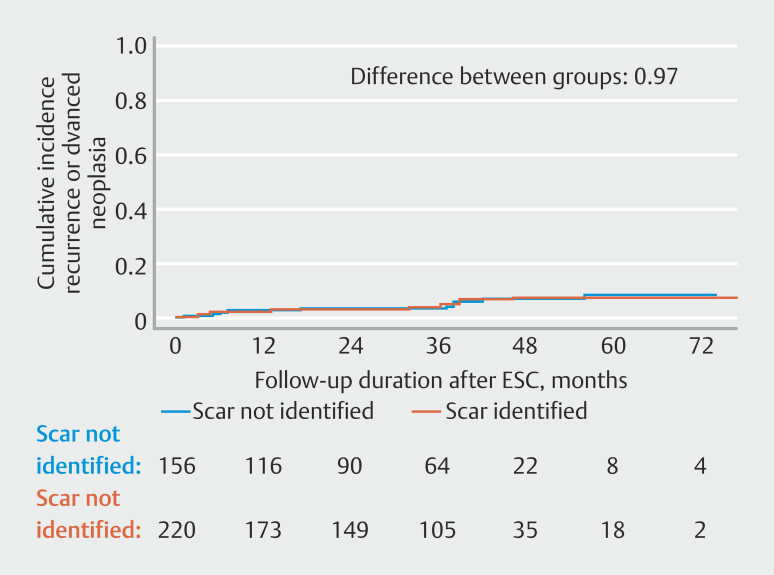
Cumulative incidence function for advanced neoplasia and recurrence at subsequent surveillance colonoscopies according to scar identification at early surveillance colonoscopy.

After exclusion of cases with recurrence (n = 35) and synchronous lesions sized ≥20 mm at ESC (n = 15), 376 cases remained in the post-ESC surveillance cohort. Of these cases, 244 (65%) underwent SC2 and 44 cases (12%) had not yet reached SC2 ( **Fig. 2s** ). The median interval between ESC and SC2 was 32 months (IQR 14–40). The overall scar identification rate at SC2 was 86 of 244 cases (35.2%; 95%CI 29.5–41.4). The scar identification rate was increased in cases with scar identification at ESC (64/148, 43.2%; 95%CI 35.5–51.3) compared with cases without scar identification at ESC (22/96, 22.9%; 95%CI 15.6–32.2; *P* = 0.001) ( **Table 9s** ).

The cumulative incidence of recurrence or same-segment advanced neoplasia at 5 years after ESC was 7.5% (95%CI 3.6–11.3) for cases with scar identification during ESC and 8.5% (95%CI 3.0–13.9) for cases without scar identification. The cumulative incidence for only recurrence during SC2 at 5 years after ESC was 5.3% (95%CI 2.0–8.5) for cases with scar identification during ESC and 5.3% (95%CI 1.4–9.2) for cases without scar identification. Our competing risk model indicated that the overall rates of recurrence and advanced neoplasia were similar between cases with and without scar identification at ESC ( *P* = 0.97). Competing events included cancelation of SC2 due to advanced age (11 vs. 10 cases), comorbidities (6 vs. 5 cases), non-CRC-related death (3 vs. 6 cases), and transfer of care to another hospital (5 vs. 1 case), for cases with and without scar identification, respectively ( Fig. 2 , **Figs. 2s** and **3s** ).

## Discussion

In this multicenter cohort study, we found an overall recurrence rate of approximately 8% at ESC after pEMR of NPCPs sized 10–20 mm. The post-pEMR scar at the primary resection site was identified in only 62% of cases, with a corresponding recurrence rate of 13% at ESC, suggesting that the true recurrence rate may lie within this range. We found no NPCP or procedural characteristics significantly associated with recurrence.

Our findings are similar to the results of a recent, single-center, retrospective study investigating recurrence after pEMR of NPCPs sized 10–19 mm compared with those sized ≥20 mm 16 . This study reported recurrence rates of 10.5% for NPCPs sized 10–19 mm and approximately 22% for lesions sized ≥20 mm, and identified lesion size as the only independent variable associated with recurrence. Notably, patients without post-pEMR scars identified were excluded, and MTA of the resection site was performed in approximately 40%. In comparison, a 2020 meta-analysis by Djinbachian et al. reported a 20% incomplete resection rate for lesions sized 10–20 mm resected with submucosal injection 6 . However, the studies in this meta-analysis were predominantly included before 2019 and reported no data about MTA use.

During our study period (2014–2021), the recurrence rate decreased over time ( **Fig. 1s** ). This decline may be attributable to the inclusion of relatively fewer cases from sites with initially higher recurrence rates later in the study period, although a reduction in recurrence rates was also observed in these high-recurrence centers. While our mixed-effects regression analysis was underpowered to detect significant associations, likely due to a low number of recurrences and high variability among endoscopists (103 in total), certain variables showed trends, including EMR expertise and MTA use. The latter may be a relevant factor, as the role of MTA in reducing recurrence is supported by studies from Klein et al. (2019) and Sidhu et al. (2021), which reported recurrence rates of 5.2% (5.4% for piecemeal resections) and 1.4%, respectively, for larger NPCPs (≥20 mm) when MTA was applied 17 18 . Additionally, a recent meta-analysis investigating recurrence rates after EMR of NPCPs >15 mm, reported an OR of 0.18 (95%CI 0.13–0.26) for recurrences with adjuvant MTA after EMR compared with EMR alone 19 . In our study, MTA was used in only 21% of cases, with recurrence rates of approximately 4% for lesions treated with MTA and 9% for lesions without MTA treatment. Although the use of MTA increased during the study period, possibly contributing to the observed decline in recurrence rates, it was not statistically significant in the regression analysis. The ESGE guidelines recommend MTA for larger NPCPs (≥20 mm); however, this recommendation has not yet been extended to NPCPs of 10–20 mm 2 . Nonetheless, hypothetically, the underlying mechanism of action should remain consistent across lesion sizes, and the potential benefits of adjuvant margin ablation should likely be extended to pEMR of NPCPs of 10–20 mm. To our knowledge, no randomized studies have yet investigated the effect of margin ablation after (piecemeal) EMR of NPCPs in this size range. Therefore, as MTA is increasingly used in daily clinical practice, future research should evaluate the efficacy of MTA not only in reducing recurrence for large (≥20 mm) lesions but also in (piecemeal) resections of smaller lesions.

For our recurrence rate, we considered both microscopic and macroscopic recurrences. Biopsies were taken in fewer than 10% of cases at ESC, with varying rationale, but predominantly yielding normal mucosa. This aligns with previous studies that have demonstrated that thorough inspection is generally sufficient for optical scar diagnosis 20 21 . Remarkably, no recurrences were identified in biopsies taken at non-teaching sites, suggesting potential challenges with optical scar diagnosis or a more conservative biopsy approach. However, these findings should be interpreted with caution due to the low biopsy rate and limited sample size.

In our study, the post-pEMR scar was only identified in approximately 60% of the colonoscopies at ESC. This moderate identification rate contrasts with scar identification rates ranging from 93% to 99.7% in studies evaluating optical assessment of post-polypectomy scars after EMR of lesions sized ≥15 or ≥20 mm, without tattooing 20 22 23 . These higher identification rates can likely be attributed to studies conducted in expert centers, with a focus on scar recognition and typically involving larger lesions. While tattoo placement was associated with higher scar identification rates in our study, increasing identification to approximately 82% from 57%, additional studies are needed to evaluate the utility of tattoo placement specifically for 10–20-mm NPCPs.

Our findings suggest that scar identification at ESC did not influence rates of recurrence or advanced neoplasia at subsequent (second) surveillance colonoscopy (SC2). This suggests that when a thorough inspection at ESC does not result in scar identification, the likelihood of a missed recurrence appears low, as similar rates of recurrence and advanced neoplasia were observed at SC2 regardless of scar identification at ESC.

Our study design precluded reliable data collection on clinically relevant outcomes such as post-colonoscopy CRC. However, prior studies indicate that while piecemeal or incomplete resections are associated with post-colonoscopy CRC, the absolute risk following incomplete resection remains low 24 25 . Given this low absolute risk and the absence of CRC or HGD at SC2 in our cohort, regardless of scar identification at ESC, the likelihood of missing clinically relevant recurrences appears minimal. Moreover, recurrences in our study were typically small and without HGD, allowing for efficient treatment using conventional endoscopic techniques, such as hot snare polypectomy with snare-tip soft coagulation or cold-forceps avulsion with adjuvant snare-tip soft coagulation, as demonstrated in a recent study 26 , thereby mitigating further potential malignant progression.

While our study reported a substantial recurrence rate, the timing and necessity of ESC should be carefully balanced against individual patient characteristics and overall use of endoscopy resources. Given that most recurrences were small and without HGD, extending the ESC interval beyond the timeframe of our study (9 months) may still allow for the detection of metachronous lesions. Furthermore, from a sustainability perspective, careful consideration of appropriateness of endoscopic procedures is considered one of the most important factors in mitigating the environmental impact of gastrointestinal endoscopy 27 . Additionally, nearly a quarter of our patients did not undergo SC2 due to factors such as comorbidities or advanced age, raising questions about the clinical benefit of performing ESC in these cases. Ultimately, the decision to perform ESC should be based on the likelihood of clinically relevant recurrence, patient-specific characteristics, and evolving demands on endoscopy services.

The strengths of this study include the multicenter approach, with both teaching and non-teaching hospitals participating. To our knowledge, this is the first multicenter study specifically addressing the recurrence rate after pEMR of NPCPs sized 10–20 mm. Additionally, as our inclusion period aligned with the implementation of the 2013 Dutch national and ESGE post-polypectomy guidelines, our real-world cohort reflects the yield of performing early repeat ESC to identify the post-pEMR scar and potential recurrences 12 13 .

Our study also has some limitations. First, our methodology of patient selection and differences in the accessibility of electronic medical records across study hospitals prevented the inclusion of patients who underwent pEMR of NPCPs sized 10–20 mm and were referred to standard surveillance recommendations for low-risk lesions 28 29 . This design may have introduced bias, as endoscopists might have chosen ESC over a longer interval for patients suspected of being at higher recurrence risk, potentially increasing the proportion of lesions with an increased recurrence risk in our study. Second, our regression analysis for recurrence was underpowered due to the low absolute incidence of recurrence in our cohort, as reflected by relatively wide confidence intervals, and should be interpreted with caution. Third, our study included NPCPs ranging from 10 to 20 mm, contrary to current ESGE and USMSTF guidelines, which adopt a 20-mm cutoff and recommend a 6-month follow-up for piecemeal resections of polyps sized ≥20 mm 2 3 28 29 . However, we included this size range to address the risk of terminal digit bias in reporting polyp sizes, particularly considering the retrospective design of our study.

In conclusion, our study found a substantial recurrence rate after pEMR of NPCPs sized 10–20 mm at ESC. Although this recurrence rate was relatively high, most recurrences were small and without advanced neoplasia. This suggests that, when deciding to perform ESC, modestly extending the surveillance interval beyond that of our study may be appropriate to detect potential metachronous lesions. Additionally, although scar identification at ESC was moderate, our SC2 findings suggest that the incidence of late recurrence or clinically relevant advanced neoplasia was comparable regardless of scar identification at ESC.

## Supplementary Material

Supplementary Material Zusatzmaterial
