# Supplementary material for: Recurrence after piecemeal hot-snare endoscopic mucosal resection of 10–20-mm nonpedunculated colorectal polyps: a multicenter cohort study
Source: Endoscopy. 2025 May 19;57(7):740–9. doi: 10.1055/a-2563-1606 (PMC12224662; doi:10.1055/a-2563-1606)
Supplement: Supplementary file 1 — Supplementary Material [file 10-1055-a-2563-1606_25990967.pdf]

Supplementary material

Recurrence after piecemeal hot-snare endoscopic mucosal resection of 10–20-mm nonpedunculated colorectal polyps: a multicenter cohort study

Michiel H.J. Maas, Yark Hazewinkel, Jochim S. Terhaar Sive Droste, Ruud W.M. Schrauwen, Adriaan C. Tan, Parweez Koehestanie, Mariëtte C.A. van Kouwen, Peter D. Siersema

Table 1s Patient, NPCP, and primary and early surveillance colonoscopy characteristics by study site.

**Patient information**	**Study site A (n = 83)**	**Study site B (n = 103)**	**Study site C (n = 58)**	**Study site D (n = 85)**	**Study site E (n = 60)**	**Overall (n = 389)**
Age, years	66.0 (61.0 – 71.0)	69.0 (64.0 – 73.0)	68.0 (62.8 – 73.0)	66.0 (59.5 – 71.0)	68.5 (64.3 – 73.8)	68.0 (63.0 – 72.0)
Sex						
Male	45/83 (54.2)	62/103 (60.2)	29/58 (50.0)	46/85 (54.1)	37/60 (61.7)	219/389 (56.3)
Female	38/83 (45.8)	41/103 (39.8)	29/58 (50.0)	39/85 (45.9)	23/60 (38.3)	170/389 (43.7)
Family history[1]						
Positive family history for CRC[2]	5/81 (6.2)	9/81 (11.1)	6/54 (11.1)	8/74 (9.4)	2/46 (4.3)	30/336 (8.9)
Lynch syndrome	4/81 (4.9)	2/81 (2.5)	1/54 (1.9)	0/74 (0)	1/46 (2.2)	8/336 (2.4)
Reason for colonoscopy						
non-FIT screening	1/83 (1.2)	2/103 (1.9)	3/58 (5.2)	1/85 (1.2)	0/60 (0)	7/389 (1.8)
FIT+ screening	27/83 (32.5)	43/103 (41.7)	17/58 (29.3)	40/85 (47.1)	32/60 (53.3)	159/389 (40.9)
Surveillance	25/83 (30.1)	34/103 (33.0)	21/58 (36.2)	23/85 (27.1)	17/60 (28.3)	120/389 (30.8)
Symptomatic	6/83 (7.2)	18/103 (17.5)	8/58 (13.8)	12/85 (14.1)	5/60 (8.3)	49/389 (12.6)
Scheduled EMR procedure	24/83 (28.9)	4/103 (3.9)	9/58 (15.5)	9/85 (10.6)	3/60 (5.0)	49/389 (12.6)
Other	0/83 (0)	2/103 (1.9)	0/58 (0)	0/85 (0)	3/60 (5.0)	5/389 (1.3)
**NPCP and procedural information**	**Study site A (n = 89)**	**Study site B (n = 111)**	**Study site C (n = 71)**	**Study site D (n = 90)**	**Study site E (n = 65)**	**Overall (n = 426)**
Lesion size, mm	15.0 (13.5 – 20.0)	15.0 (12.0 – 20.0)	15.0 (12.0 – 20.0)	15.0 (15.0 – 20.0)	15.0 (13.5 – 20.0)	15.0 (12.8 – 20.0)
Location of primary lesion						
Proximal colon[3]	69/89 (77.5)	76/111 (68.5)	60/71 (84.5)	80/90 (88.9)	57/65 (87.7)	342/426 (80.3)
Distal colon	20/89 (22.5)	35/111 (31.5)	11/71 (15.5)	10/90 (11.1)	8/65 (12.3)	84/426 (19.7)
pEMR performed by						
Senior endoscopist	54/89 (60.7)	93/111 (83.8)	65/71 (91.5)	90/90 (100)	65/65 (100)	367/426 (86.2)
Junior endoscopist	35/89 (39.3)	14/111 (12.6)	0/71 (0)	0/90 (0)	0/65 (0)	49/426 (11.5)
Nurse endoscopist	0/89 (0)	4/111 (3.6)	6/71 (8.5)	0/90 (0)	0/65 (0)	10/426 (2.3)
EMR expert[4]	7/89 (7.9)	52/111 (46.8)	15/71 (21.1)	15/90 (16.7)	9/65 (13.8)	98/426 (23.0)
Number of pieces per pEMR[5]	3 (2 – 4)	2 (2 – 2)	2 (2 – 2)	3 (2 – 3)	2 (2 – 3)	2 (2 – 3)

Thieme

Intraprocedural bleeding	18/89 (20.2)	12/111 (10.8)	3/71 (4.2)	13/90 (14.4)	9/65 (13.8)	55/426 (12.9)
Post-EMR clip placement	31/89 (34.8)	19/111 (17.1)	19/71 (26.8)	26/90 (28.9)	21/65 (32.3)	116/426 (27.2)
Adjuvant therapy used						
STSC	25/89 (28.1)	5/111 (4.5)	9/71 (12.7)	18/90 (20.0)	10/65 (15.4)	67/426 (15.7)
APC	3/89 (3.4)	13/111 (11.7)	3/71 (4.2)	3/90 (3.3)	3/65 (4.6)	25/426 (5.9)
Tattoo placed	18/89 (20.2)	15/111 (13.5)	5/71 (7.0)	23/90 (25.6)	15/65 (23.1)	76/426 (17.8)
pEMR performing endoscopists	35/103 (34.0)	24/103 (23.3)	14/103 (13.6)	13/103 (12.6)	17/103 (16.5)	103/103 (100)
Median pEMR per endoscopist	1 (1.0 – 3.0)	3 (1.0 – 5.0)	4.5 (1.0 – 7.3)	5 (2.5 – 6.5)	3 (1.5 – 6.0)	3.0 (1.0 – 6.0)
**ESC information**	**Study site A (n = 89)**	**Study site B (n = 111)**	**Study site C (n = 71)**	**Study site D (n = 90)**	**Study site E (n = 65)**	**Overall (n = 426)**
Time to ESC, weeks	30 (25.5 – 34)	26 (21.0 – 32.0)	27.0 (23.0 – 31.0)	26.0 (24.0 – 29.0)	27.0 (24.0 – 30.0)	27.0 (23.8 – 31.0)
ESC performed by						
Senior endoscopist	29/89 (32.6)	86/111 (77.5)	47/111 (66.2)	90/90 (100)	62/65 (95.4)	314/426 (73.7)
Junior endoscopist	60/89 (67.4)	16/111 (14.4)	0/111 (0)	0/90 (0)	0/65 (0)	76/426 (17.8)
Nurse endoscopist	0/89 (0)	9/111 (8.1)	24/71 (33.8)	0/90 (0)	3/65 (4.6)	36/426 (8.5)
EMR expert[4]	8/89 (9.0)	38/111 (34.2)	9/71 (12.7)	24/90 (26.7)	18/65 (27.7)	97/426 (22.8)
BBPS of segment of post-pEMR site[6]						
3	53/78 (67.9)	45/70 (64.3)	47/67 (70.1)	61/76 (80.3)	42/62 (67.6)	248/353 (70.3)
2	24/78 (30.8)	23/70 (32.9)	18/67 (26.9)	14/76 (18.4)	19/62 (30.6)	98/353 (27.8)
1	0/78 (0)	2/79 (2.9)	2/67 (3.0)	1/76 (1.3)	1/62 (1.6)	6/353 (1.7)
0	1/78 (1.3)	0/70 (0)	0/67 (0)	0/76 (0)	0/62 (0)	1/353 (0.3)
Biopsy of post-pEMR scar	7/56 (12.5)	7/89 (11.9)	3/40 (7.5)	5/69 (7.2)	1/38 (2.6)	23/262 (8.8)

Data are presented as n/N (%), or median (IQR). APC = argon plasma coagulation, BBPS = Boston Bowel Preparation Score, CRC = colorectal cancer, ESC = early surveillance colonoscopy, ESD = endoscopic submucosal dissection, FIT = fecal immunochemical test, NPCP = non-pedunculated colorectal polyp, pEMR = piecemeal endoscopic mucosal resection, STSC = snare-tip soft coagulation.

[1]Family history was not reported in 53 patients.

[2]Defined as one first-degree relative diagnosed before age 50, or one first-degree relative diagnosed between 50-70 and one second-degree relative diagnosed before 70, or two or more first-degree relatives diagnosed between 50-70.

[3]Proximal colon was defined as all segments proximal to the splenic flexure.

[4]EMR expert was defined as an endoscopist with a minimum of 30 performed EMRs or ESDs per year, on average.

[5]Median calculated on the total number of cases (n = 93) in which number of pieces per resection was reported.

[6]73 cases missed BBPS data at ESC.

Table 2s Patient, NPCP, and primary and surveillance colonoscopy characteristics by year of primary resection.

**Patient information**	**2014 (n = 13)**	**2015 (n = 34)**	**2016 (n = 38)**	**2017 (n = 70)**	**2018 (n = 82)**	**2019 (n = 60)**	**2020 (n = 63)**	**2021 (n = 29)**	**Overall (n = 389)**
Age, years	67 (63 – 76	67 (64 – 71)	69 (63 – 71)	67 (62 – 70)	66 (61 – 72)	69 (63 – 73)	67 (63 – 73)	70 (64 – 75)	68 (63 – 72)
Sex									
Male	7/13 (54)	22/34 (65)	24/38 (63)	39/70 (56)	42/82 (51)	36/60 (60)	30/63 (52)	19/29 (66)	219/389 (56)
Female	6/13 (46)	12/34 (35)	14/38 (37)	31/70 (56)	40/82 (49)	24/60 (40)	33/63 (48)	10/29 (35)	170/389 (44)
Family history[1]									
Positive family history for CRC[2]	1/12 (8)	1/31 (3)	5/31 (16)	2/61 (3)	12/70 (17)	5/47 (11)	2/59 (3)	2/25 (8)	30/336 (8.9)
Lynch syndrome	0/12 (0)	0/31 (0)	0/31 (0)	2/61 (3)	1/70(1)	3/47 (6)	1/59 (2)	1/25 (4)	8/336 (2.4)
Reason for colonoscopy									
non-FIT screening	0/13 (0)	0/34 (0)	0/38 (0)	1/70 (1)	3/82 (4)	2/60 (3.3)	0/63 (0)	1/29 (3)	7/389 (2)
FIT+ screening	6/13 (46)	20/34 (59)	12/38 (32)	39/70 (56)	30/82 (37)	21/60 (35)	25/63 (40)	6/29 (21)	159/389 (41)
Surveillance	0/13 (0)	8/34 (24)	10/38 (26)	15/70 (21)	29/82 (35)	25/60 (42)	21/63 (33)	12/29 (41)	120/389 (31)
Symptomatic	4/13 (31)	1/34 (3)	9/38 (24)	7/70 (10)	12/82 (15)	2/60 (3.3)	9/63 (14)	5/29 (17)	49/389 (13)
Scheduled EMR procedure	3/13 (23)	4/34 (12)	7/38 (18)	8/70 (11.4)	7/82 (9)	7/60 (12)	8/63 (13)	5/29 (17)	49/389 (13)
Other	0/13 (0)	1/34 (3)	0/38 (0)	0/70 (0)	1/82 (1)	3/60 (5)	0/63 (0)	0/29 (0)	5/389 (1)
**NPCP and procedural information**	**2014 (n = 13)**	**2015 (n = 37)**	**2016 (n = 41)**	**2017 (n = 76)**	**2018 (n = 90)**	**2019 (n = 68)**	**2020 (n = 69)**	**2021 (n = 32)**	**Overall (n = 426)**
Lesion size, mm	12 (12 – 17)	15 (14 – 20)	15 (14 – 20)	15 (15 – 20)	15 (13 – 20)	15 (12 – 20)	15 (15 - 20)	16 (12 – 20)	15 (13 – 20)
Location of primary lesion									
Proximal colon[3]	11/13 (85)	23/37 (62)	33/41 (81)	59/76 (78)	76/90 (84)	55/68 (81)	60/69 (87)	25/32 (78)	342/426 (80)
Distal colon	2/13 (15)	14/37 (38)	8/41 (20)	17/76 (22)	14/90 (16)	13/68 (19)	9/69 (13)	7/32 (22)	84/426 (20)
pEMR performed by									
Senior endoscopist	12/13 (92)	35/37 (95)	33/41 (81)	62/76 (82)	81/90 (90)	55/68 (81)	61/69 (88)	28/32 (88)	367/426 (86)
Junior endoscopist	1/13 (8)	2/37 (5)	8/41 (20)	14/76 (18)	7/90 (8)	9/68 (13)	6/69 (9)	2/32 (6)	49/426 (12)
Nurse endoscopist	0/13 (0)	0/37 (0)	0/41 (0)	0/76 (0)	2/90 (2)	4/68 (6)	2/69 (3)	2/32 (6)	10/426 (2)
EMR expert[4]	7/13 (54)	10/37 (27)	8/41 (20)	15/76 (20)	19/90 (21)	14/68 (21)	19/69 (28)	6/32 (19)	98/426 (23)

Number of pieces per pEMR[5]	2 (2 – .)	3 (2 – 4)	3 (2 – 4)	2 (2 – 3)	2 (2 – 3)	3 (2 – 4)	2 (2 – 3)	2 (2 – 3)	2 (2 – 3)
Intraprocedural bleeding	3/13 (23)	7/37 (19)	7/41 (17)	7/76 (8)	9/90 (10)	6/68 (9)	13/69 (20)	3/32 (9)	55/426 (13)
Post-EMR clip placement	6/13 (46)	12/37 (32)	13/41 (32)	17/76 (22)	22/90 (24)	14/68 (21)	25/69 (35)	8/32 (25)	116/426 (27)
Adjuvant therapy used									
STSC	0/13 (0)	5/37 (14)	5/41 (12)	12/76 (16)	12/90 (13)	14/68 (21)	12/69 (17)	7/32 (22)	67/426 (16)
APC	4/13 (31)	1/37 (3)	3/41 (7)	3/76 (4)	7/90 (8)	4/68 (6)	2/69 3)	1/32 (3)	25/426 (6)
Tattoo placed	2/13 (15)	7/37 (19)	7/41 (17)	5/76 (7)	18/90 (20)	21 (31)	9/69 (13)	7/32 (22)	76/426 (18)
**ESC information**	**2014 (n = 13)**	**2015 (n = 37)**	**2016 (n = 41)**	**2017 (n = 76)**	**2018 (n = 90)**	**2019 (n = 68)**	**2020 (n = 69)**	**2021 (n = 32)**	**Overall (n = 426)**
Time to ESC, weeks	26 (24 – 32)	26 (20 – 29)	26 (23 – 33)	26 (22 – 31)	27 (23 – 31)	28 (26 – 32)	28 (24 – 31)	29 (25 – 33)	27 (24 – 31)
ESC performed by									
Senior endoscopist	9/13 (69)	27/37 (73)	26/41 (63)	46/76 (61)	76/90 (84)	52/68 (77)	53/69 (77)	25/32 (78)	314/426 (74)
Junior endoscopist	4/13 (31)	10/37 (27)	13/41 (32)	25/76 (33)	8/90 (9)	10/68 (15)	3/69 (4)	3/32 (9)	76/426 (18)
Nurse endoscopist	0/13	0/27 (0)	2/41 (5)	5/76 (7)	6/90 (7)	6/68 (9)	13/69 (19)	4/32 (13)	36/426 (9)
EMR expert[4]	7/13 (54)	8/37 (22)	3/41 (7)	11/76 (15)	14/90 (16)	19/68 (28)	27/69 (39)	8/32 (25)	97/426 (23)
Scar identification	8/13 (62)	23/37 (62)	26/41 (63)	46/76 (61)	54/90 (60)	39/68 (57)	45/69 (65)	21/32 (66)	262/426 (62)
Recurrence	1/13 (8)	7/37 (19)	6/41 (15)	9/76 (12)	4/90 (4)	4/68 (6)	4/69 (6)	0/32 (0)	35/426 (8)
BBPS of segment of post-pEMR site[6]									
3	3/4 (75)	19/26 (73)	18/28 (64)	48/62 (77)	51/79 (65)	41/60 (68)	50/65 (77)	18/29 (62)	248/353 (70)
2	1/4 (25)	6/26 (23)	10/28 (36)	14/62 (23)	27/79 (34)	18/60 (30)	14/65 (22)	8/29 (28)	98/353 (28)
1	0/4 (0)	0/26 (0)	0/28 (0)	0/62	1/79 (1)	1/60 (2)	1/69 (2)	3/29 (10)	6/353 (2)
0	0/4 (0)	1/26 (4)	0/28 (0)	0/62	0/79 (0)	0/60 (0)	0/69 (0)	0/29 (0)	1/353 (0.3)
Biopsy of post-pEMR scar	0/8 (0)	6/23 (26)	2/26 (8)	3/46 (7)	5/54 (9)	2/39 (5)	3/45 (7)	2/21 (10)	23/262 (9)

Data are presented as n/N (%), or median (IQR). APC = argon plasma coagulation, BBPS = Boston Bowel Preparation Score, CRC = colorectal cancer, ESC = early surveillance colonoscopy, ESD = endoscopic submucosal dissection, FIT = fecal immunochemical test, NPCP = non-pedunculated colorectal polyp, pEMR = piecemeal endoscopic mucosal resection, STSC = snare-tip soft coagulation.

[1]Family history was not reported in 53 patients.

[2]Defined as one first-degree relative diagnosed before age 50, or one first-degree relative diagnosed between 50-70 and one second-degree relative diagnosed before 70, or two or more first-degree relatives diagnosed between 50-70.

[3]Proximal colon was defined as all segments proximal to the splenic flexure.

[4]EMR expert was defined as an endoscopist with a minimum of 30 performed EMRs or ESDs per year, on average.

[5]Median calculated on the total number of cases (n = 93) in which number of pieces per resection was reported.

[6]73 cases missed BBPS data at ESC.

Table 3s Patient, NPCP, and primary and surveillance colonoscopy characteristics by year of primary resection categorized.

**Patient information**	**2014 – 2018 (n = 237)**	**2019 – 2021 (n = 152)**	**p value**
Age, years	67.0 (62.0 – 71.0)	68.0 (63.0 – 73.0)	.45
Sex, male	134/237 (56.5)	85/152 (55.9)	.90
Family history[1]			.24
Positive family history for CRC[2]	21/205 (10.2)	9/131 (6.9)	
Lynch syndrome	3/205 (1.5)	5/131 (3.8)	
Reason for colonoscopy			.11
non-FIT screening	4/237 (1.7)	3/152 (2.0)	
FIT+ screening	107/237 (45.1)	52/152 (34.2)	
Surveillance	62/237 (26.2)	58/152 (38.2)	
Symptomatic	33/237 (13.9)	16/152 (10.5)	
Scheduled EMR procedure	29/237 (12.2)	20/152 (13.2)	
Other	2/237 (0.8)	3/152 (2.0)	
**NPCP information**	**2014 – 2018 (n = 257)**	**2019 – 2021 (n = 169)**	
Lesion size, mm	15.0 (13.0 – 20.0)	15.0 (12.0 – 20.0)	.86
Location of primary lesion			.28
Proximal colon[3]	202/257 (78.6)	140/169 (82.8)	
Distal colon	55/257 (21.4)	29/169 (17.2)	
pEMR performed by			.026
Senior endoscopist	223/257 (86.8)	144/169 (85.2)	
Junior endoscopist	32/257 (12.5)	17/169 (10.1)	
Nurse endoscopist	2/257 (0.8)	8/169 (4.7)	
EMR expert[4]	59/257 (23.0)	39/169 (23.1)	.98
Number of pieces per pEMR[5]	2.0 (2.0 – 3.0)	2.0 (2.0 – 3.0)	.99
Intraprocedural bleeding	33/257 (12.8)	22/169 (13.0)	.96
Post-EMR clip placement	70/257 (27.2)	46/169 (27.2)	.997
Adjuvant therapy used			
STSC	34/257 (13.2)	33/169 (19.5)	.081
APC	18/257 (7.0)	7/169 (4.1)	.22
Tattoo placed	39/257 (15.2)	37/169 (21.9)	.076
**ESC information**	**2014 – 2018 (n = 257)**	**2019 – 2021 (n = 169)**	

Time to ESC, weeks	26.0 (22.0 – 31.0)	28.0 (25.0 – 32.0)	.003
ESC performed by			<.001
Senior endoscopist	184/257 (71.6)	130/169 (76.9)	
Junior endoscopist	60/257 (23.3)	16/169 (9.5)	
Nurse endoscopist	13/257 (5.1)	23/169 (13.6)	
EMR expert[4]	43/257 (16.7)	54/169 (32.0)	<.001
Recurrence	27/257 (10.5) [7.3 – 14.9]	8/169 (4.7) [2.4 – 9.1]	.034
Scar identification	157/257 (61.1) [55.0 – 66.8]	105/169 (62.1) [54.6 – 69.1]	.83

Data are presented as n/N (%), or median (IQR). APC = argon plasma coagulation, BBPS = Boston Bowel Preparation Score, CRC = colorectal cancer, ESC = early surveillance colonoscopy, ESD = endoscopic submucosal dissection, FIT = fecal immunochemical test, NPCP = non-pedunculated colorectal polyp, pEMR = piecemeal endoscopic mucosal resection, STSC = snare-tip soft coagulation.

[1]Family history was not reported in 53 patients.

[2]Defined as one first-degree relative diagnosed before age 50, or one first-degree relative diagnosed between 50-70 and one second-degree relative diagnosed before 70, or two or more first-degree relatives diagnosed between 50-70.

[3]Proximal colon was defined as all segments proximal to the splenic flexure.

[4]EMR expert was defined as an endoscopist with a minimum of 30 performed EMRs or ESDs per year, on average.

[5]Median calculated on the total number of cases (n = 93) in which number of pieces per resection was reported.

Table 4s Overall recurrence rates, recurrence rates per adenomatous or serrated lesions, and scar identification rates at early surveillance colonoscopy by study site.

	Study site A (n = 89)	Study site B (n = 111)	Study site C (n = 71)	Study site D (n = 90)	Study site E (n = 65)	Overall (n = 426)
Overall recurrence	12/89 (13.5) [7.9 – 22.1]	12/111 (10.8) [6.3 – 18.0]	4/71 (5.6) [2.2 – 13.6]	4/90 (4.4) [1.7 – 10.9]	3/65 (4.6) [1.6 – 12.7	35/426 (8.2) [6.0 – 11.2]
Recurrence of adenomas	11/63 (17.5) [10.0 – 28.6]	8/74 (10.8) [5.6 – 19.9]	2/39 (5.1) [1.4 – 16.9]	2/60 (3.3) [0.9 – 11.4]	3/53 (5.7) [1.9 – 15.4]	26/289 (9.0) [6.2 – 12.9]
Recurrence of serrated lesions[1]	1/22 (4.5) [0.8 – 21.8[	4/33 (12.1) [4.8 – 27.3]	2/31 (6.5) [1.8 – 20.7]	2/30 (6.7) [1.8 – 21.3]	0/12 (0) [0 – 24.2]	9/128 (7.0) [3.7 – 12.8]
Recurrence of other NPCPs[2]	0/4 (0)	0/4 (0)	(0/1) (0)	…	…	0/9 (0) [0.0 – 29.9]
Median recurrence size, mm[3]	6.0 (1.0 – 8.0)	1.5 (1.0 – 6.0)	8.0 (5.0 – .)	3.0 (3.0 – 3.0)	4.5 (3.0 – .)	5.0 (1.0 – 7.0)
Identification of post-pEMR scar	56/89 (62.9) [52.5 – 72.2]	59/111 (53.2) [71.8 – 86.5]	40/71 (56.3) [44.8 – 67.3]	69/90 (76.7) [67.0 – 84.2]	38/65 (58.6) [46.3 – 69.6]	262/426 (61.5) [56.8 – 66.0]
Scar identification of tattooed resection sites	14/18 (77.8) [54.8 – 91.0]	13/15 (86.7) [62.1 – 92.3]	4/5 (80.0) [37.6 – 96.4]	21/23 (91.3) [73.2 – 97.6]	10/15 (66.7) [41.7 – 84.8]	62/76 (81.6) [71.4 – 88.7]
Scar identification of non-tattooed resection sites	42/71 (59.2) [47.5 – 69.8]	46/96 (47.9) [38.2 – 57.8]	36/66 (54.5) [42.6 – 66.0]	48/67 (71.6) [59.9 – 81.0]	28/50 (56.0) [42.3 – 63.8]	200/350 (57.1) [51.9 – 62.2]
Overall recurrence of cases with scar identification	12/56 (21.4) [12.7 – 33.8]	12/59 (20.3) [12.0 – 32.3]	4/40 (10.0) [4.0 – 23.1]	4/69 (5.8) [2.3 – 14.0]	3/38 (7.9) [2.7 – 20.8]	35/262 (13.4) [9.8 – 18.0]

Data are presented as n/N (%), median with (IQR), or [95% CI]. [95% CI] was calculated with Wilson Score Interval. CI = confidence interval, IQR = interquartile range, NPCP = non-pedunculated colorectal polyp.

[1]Serrated lesions included sessile serrated lesions (n=105), hyperplastic polyps (n=17), and traditional serrated adenomas (n=6).

[2]Other lesions (n=9) included NPCPs with indefinite or missing histopathologic diagnosis.

[3]Size of recurrence was reported in 19 of 30 cases with macroscopic recurrence.

Table 5s Recurrence rates of serrated lesions at early surveillance colonoscopy.

	Recurrence rate
Sessile serrated lesion without dysplasia	6/92 (6.5)<br>[3.0 – 13.5]
Sessile serrated lesion with dysplasia	2/13 (15.4)<br>[4.3 – 42.2]
Hyperplastic polyp	1/17 (5.9)<br>[1.0 – 27.0]
Traditional serrated adenoma	0/6 (0)<br>[0 – 39.0]

Data are presented as n/N (%) or [95% CI]. [95% CI] was calculated with Wilson Score Interval
CI = Confidence interval.

Table 6s Reasons for tattoo placement during the primary colonoscopy by study site.

	**Study site A (n = 18)**	**Study site B (n = 15)**	**Study site C (n = 5)**	**Study site D (n = 23)**	**Study site E (n = 15)**	**Overall (N = 76)**
Concerns about future site identification	11/18 (61)	6/15 (40)	1/5 (20)	6/23 (26)	6/15 (40)	30/76 (40)
Concerns about malignancy[1]	3/18 (17)	1/15 (7)	0/5 (0)	0/23 (0)	1/15 (7)	5/76 (7)
Concerns about recurrence[2]	3/18 (6)	2/15 (13)	0/5 (0)	5/23 (22)	0/15 (0)	10/76 (13)
Reason unknown	1/18 (6)	6/15 (40)	4/5 (80)	12/23 (52)	8/15 (41)	31/76 (41)

Data are presented as n/N (%).
[1]1 out of 5 tattooed cases had high-grade dysplasia in the primary resection, without recurrence.
[2]1 out of 10 tattooed cases had local recurrence at ESC.

Table 7s Reasons for taking biopsies of the scar during ESC by study site.

	**Study site A (n = 7)**	**Study site B (n = 7)**	**Study site C (n = 3)**	**Study site D (n = 5)**	**Study site E (n = 1)**	**Overall (N = 23)**
Scar without macroscopic recurrence, reason unknown[1]	6/7 (86)	1/7 (14)	3/3 (100)	1/5 (20)	...	11/23 (48)
Scar without macroscopic recurrence, precautionary	1/7 (14)	2/7 (29)	...	1/5 (20)	1/1 (100)	5/23 (22)
HGD in primary lesion	...	1/7 (14)	...	1/5 (20)	...	2/23 (9)
Biopsy required due to other study	...	...	...	2/5 (40)	...	2/23 (9)
Suspected recurrence[2]	...	2/7 (29)	...	...	...	2/23 (9)
Clip artefact[3]	...	1/7 (14)	...	...	...	1/23 (4)

Data are presented as n/N (%). HGD = high-grade dysplasia.
[1]3 out of 7 cases with an unknown reason had microscopic recurrence.
[2]1 out of 2 cases with suspected recurrence had microscopic recurrence.
[3]1 out of 1 case with a clip artefact had microscopic recurrence.

Table 8s Histopathological diagnosis of lesions at the identified scar during ESC by study site.

**Macroscopic lesions**	**Study site A (n = 15)**	**Study site B (n = 12)**	**Study site C (n = 12)**	**Study site D (n = 12)**	**Study site E (n = 4)**	**Overall (N = 55)**
Adenoma with low-grade dysplasia	8/15 (53)	8/12 (66.7)	2/12 (16.7)	2/12 (16.7)	3/4 (75.0)	23/56 (42)
Adenoma with high-grade dysplasia	...	...	...	...	...	...
Sessile serrated lesion without dysplasia	1/15 (7)	1/12 (8.3)	2/12 (16.7)	1/12 (8.3)	...	5/55 (9)
Sessile serrated lesion with dysplasia	...	...	...	...	...	...
Hyperplastic polyp	...	1/12 (8.3)	...	1/12 (8.3)	...	2/55 (4)
Traditional serrated adenoma	...	...	...	...	...	...
Normal mucosa	4/15 (27)	2/12 (16.7)	8/12 (66.7)	8/12 (66.7)	1/4 (25.0)	23/55 (42)
Other[1]	2/15 (13)	...	...	...	...	2/55 (4)
Primary NPCP 10-19mm	**(n = 11)**	**(n = 9)**	**(n = 7)**	**(n = 9)**	**(n = 2)**	**(n = 38)**
Adenoma	7/11 (64)	7/9 (78)	1/7 (14)	1/9 (11)	2/2 (100)	18/38 (47)
SSL	...	1/9 (11)	2/7 (29)	1/9 (11)	...	4/38 (11)
Hyperplastic	...	1/9 (11)	...	1/9 (11)	...	2/38 (5)
Normal mucosa	3/11 (27)	...	4/7 (57)	6/9 (67)	...	13/38 (34)
Other[1]	1/11 (9)	...	...	...	...	1/38 (3)
Primary NPCP 20mm	**(n = 4)**	**(n = 3)**	**(n = 5)**	**(n = 3)**	**(n = 2)**	**(n = 17)**
Adenoma	1/4 (25)	1/3 (33)	1/5 (20)	1/3 (33)	1/2 (50)	5/15 (29)
SSL	1/4 (25)	...	...	...	...	1/15 (6)
Hyperplastic	...	...	...	...	...	...
Normal mucosa	1/4 (25)	2/3 (67)	4/5 (80)	2/3 (33)	1/2 (50)	10/17 (58)
Other[1]	1/4 (25)	...	...	...	...	1/17 (6)
**Scar biopsy**	**Study site A (n = 7)**	**Study site B (n = 7)**	**Study site C (n = 3)**	**Study site D (n = 5)**	**Study site E (n = 1)**	**Overall (N = 23)**
Adenoma with low-grade dysplasia	3/7 (43)	2/7 (29)	...	...	...	5/23 (22)

Adenoma with high-grade dysplasia	...	...	...	...	...	...
Sessile serrated lesion without dysplasia	...	...	...	...	...	...
Sessile serrated lesion with dysplasia	...	...	...	...	...	...
Hyperplastic polyp	...	...	...	...	...	...
Traditional serrated adenoma	...	...	...	...	...	...
Normal mucosa	4/7 (57)	5/7 (71)	3/3 (100)	5/5 (100)	1/1 (100)	18/23 (78)
Other	...	...	...	...	...	...
Primary NPCP 10-19mm	**(n = 4)**	**(n = 4)**	**(n =3)**	**(n = 0)**	**(n = 0)**	**(n = 11)**
Adenoma	1/4 (25)	2/4 (50)	...	...	...	3/11 (27)
SSL	...	...	...	...	...	...
Hyperplastic	...	...	...	...	...	...
Normal mucosa	3/4 (75)	2/4 (50)	3/3 (100)	...	...	8/11 (73)
Other	...	...	...	...	...	...
Primary NPCP 20mm	**(n = 3)**	**(n = 3)**	**(n = 0)**	**(n = 5)**	**(n = 1)**	**(n = 12)**
Adenoma	2/3 (67)	...	...	...	...	2/12 (17)
SSL	...	...	...	...	...	...
Hyperplastic	...	...	...	...	...	...
Normal mucosa	1/3 (33)	3/3 (100)	...	5/5 (100)	1/1 (100)	10/12 (83)
Other	...	...	...	...	...	...

Data are presented as n/N (%). NPCP = non-pedunculated colorectal polyp, SSL = sessile serrated lesion.
[1]Other lesions (n=2) included lesions with a diagnosis of ischemic tissue (n=1) and indefinite result (n=1).

Table 9s Second surveillance colonoscopy (SC2) outcomes of cases without recurrence or synchronous large (≥20 mm) lesions at ESC.

	Overall SC2 (n = 244)	Scar identified at ESC (n = 148)	Scar not identified at ESC (n = 96)	p value
Interval ESC to SC2, months	32.0 (14.0 – 40.0)	32.5 (14.0 – 42.0)	30.5 (13.3 – 38.0)	.13
Scar identification at SC2	86/244 (35.2) [29.5 – 41.4]	64/148 (43.2) [35.5 – 51.3]	22/96 (22.9) [15.6 – 32.2]	.001
Scar identification of tattooed resection sites[1]	22/41 (53.7) [38.7 – 67.9]	19/31 (61.3) [43.8 – 76.3]	3/10 (30.0) [10.8 – 60.3]	.084
Scar identification of non-tattooed resection sites[2]	64/203 (31.5) [25.5 – 38.2]	45/117 (38.5) [30.1 – 47.5]	19/86 (22.1) [14.6 – 31.9]	.013
Recurrence or AN at SC2	24/244 (9.8) [6.7 – 14.2]	14/148 (9.5) [5.7 – 15.3]	10/96 (10.4) [5.8 – 18.1]	.86
Recurrence	17/24 (70.8) [50.8 – 85.1]	10/14 (71.4) [45.4 – 88.3]	7/10 (70.0) [39.7 – 89.2]	.94
AN	8/24 (33.3) [18.0 – 53.3]	4/14 (28.6) [11.7 – 54.6]	4/10 (40.0) [16.8 – 68.7]	.56
Median recurrence size, mm[4]	6.5 (3.3 – 10.0)	6.5 (2.8 – 10.0)	7.5 (3.5 – 11.3)	.74
BBPS of segment of post-pEMR site[5]				.26
3	175/236 (74.2)	112/144 (77.8)	63/92 (68.5)	
2	57/236 (24.2)	30/144 (20.8)	27/92 (29.3)	
1	4/236 (1.7)	2/144 (1.4)	2/92 (2.2)	
0	0/236 (0)	0/144 (0)	0/92 (0)	

Data are presented as n/N (%), median with (IQR), or [95% CI]. [95% CI] was calculated with Wilson Score Interval. AN = Advanced neoplasia (≥10mm lesion or contained high-grade dysplasia), BBPS = Boston Bowel Preparation Score, CI = confidence interval, ESC = early surveillance colonoscopy, IQR = interquartile range, pEMR = piecemeal endoscopic mucosal resection.

[1]41 of 76 cases with placement of a tattoo during primary resection underwent SC2.

[2]203 of 350 cases without placement of a tattoo during primary resection underwent SC2.

[3]AN was defined as ≥10mm (n=8), or with high-grade dysplasia (n=0).

[4]14 of 15 cases of recurrence reported lesion size.

[5]8 cases missed BBPS at SC2.

Fig.1s Recurrence rate and number of included pEMR resections by year and by site.

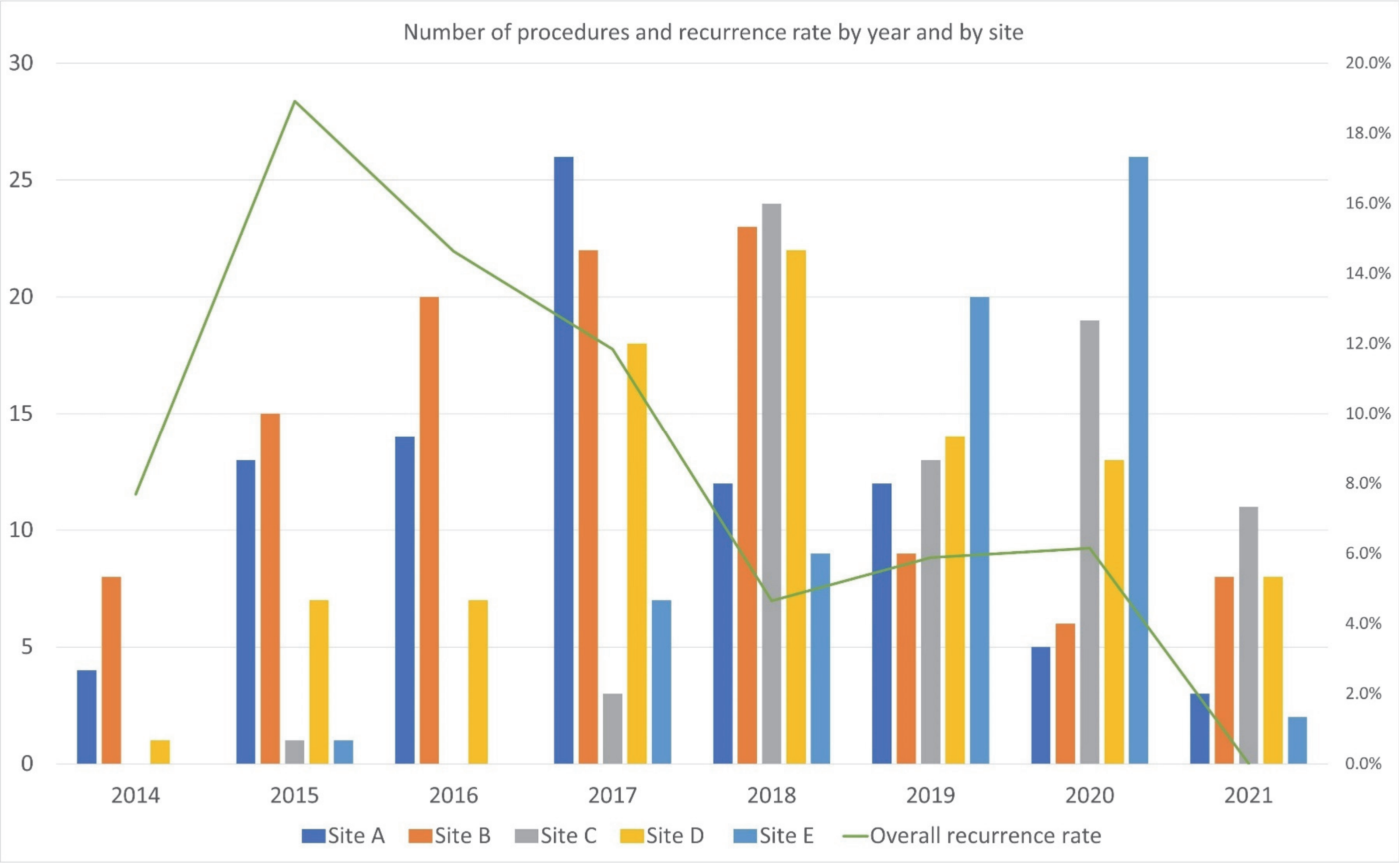

Fig. 2 Flow chart of follow-up after ESC.

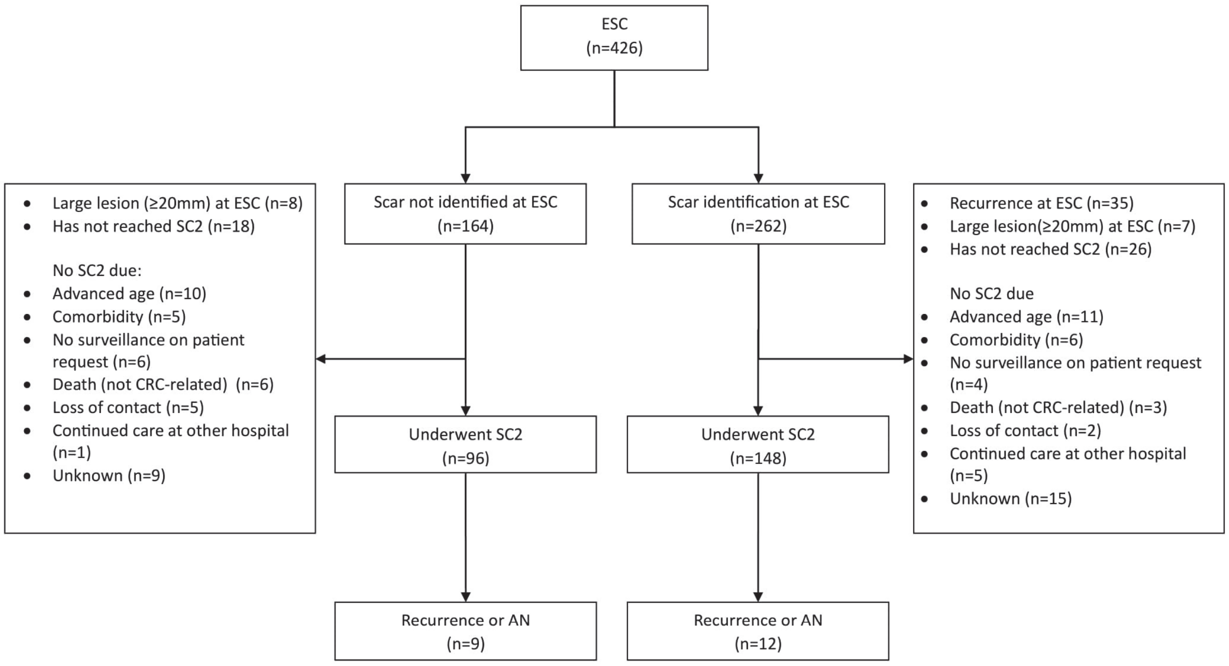

Fig. 3s Cumulative incidence for recurrence by scar identification at ESC.

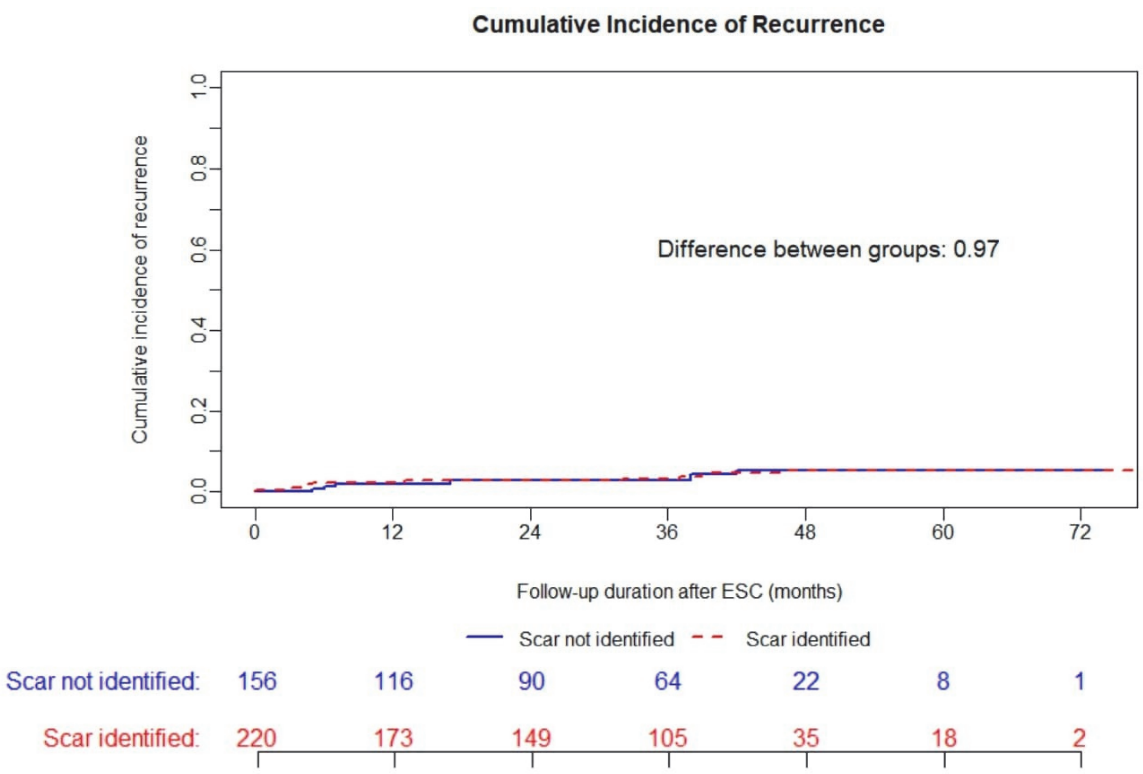

Endoscopy | DOI 10.1055/a-2563-1606 | © The Author(s).
